# *"They think we're OK and we know we're not"*. A qualitative study of asylum seekers' access, knowledge and views to health care in the UK

**DOI:** 10.1186/1472-6963-7-75

**Published:** 2007-05-30

**Authors:** Catherine A O'Donnell, Maria Higgins, Rohan Chauhan, Kenneth Mullen

**Affiliations:** 1 General Practice and Primary Care, Division of Community-based Sciences, University of Glasgow, 1 Horselethill Road, Glasgow G12 9LX, UK; 2 Psychological Medicine, Division of Community-based Sciences, University of Glasgow, Academic Centre, Gartnavel General Hospital, 1055 Great Western Road, Glasgow G12 0XH, UK

## Abstract

**Background:**

The provision of healthcare for asylum seekers is a global issue. Providing appropriate and culturally sensitive services requires us to understand the barriers facing asylum seekers and the facilitators that help them access health care. Here, we report on two linked studies exploring these issues, along with the health care needs and beliefs of asylum seekers living in the UK.

**Methods:**

Two qualitative methods were employed: focus groups facilitated by members of the asylum seeking community and interviews, either one-to-one or in a group, conducted through an interpreter. Analysis was facilitated using the Framework method.

**Results:**

Most asylum seekers were registered with a GP, facilitated for some by an Asylum Support nurse. Many experienced difficulty getting timely appointments with their doctor, especially for self-limiting symptoms that they felt could become more serious, especially in children. Most were positive about the health care they received, although some commented on the lack of continuity. However, there was surprise and disappointment at the length of waiting times both for hospital appointments and when attending accident and emergency departments. Most had attended a dentist, but usually only when there was a clinical need. The provision of interpreters in primary care was generally good, although there was a tension between interpreters translating verbatim and acting as patient advocates. Access to interpreters in other settings, e.g. in-patient hospital stays, was problematic. Barriers included the cost of over-the-counter medication, e.g. children's paracetamol; knowledge of out-of-hours medical care; and access to specialists in secondary care. Most respondents came from countries with no system of primary medical care, which impacted on their expectations of the UK system.

**Conclusion:**

Most asylum seekers were positive about their experiences of health care. However, we have identified issues regarding their understanding of how the UK system works, in particular the role of general practitioners and referral to hospital specialists. The provision of an Asylum Support nurse was clearly a facilitator to accessing primary medical care. Initiatives to increase their awareness and understanding of the UK system would be beneficial. Interpreting services also need to be developed, in particular their role in secondary care and the development of the role of interpreter as patient advocate.

## Background

In 2005, there were 336,100 applications for refugee status across Europe, Canada, USA, Australia and New Zealand [1]. Although the number of applications has been decreasing since 2000, asylum seekers and those granted refugee status represent a substantial minority ethnic population in those countries. According to both UNHCR [1] and the UK Government [2], the UK received approximately 30,500 applications for asylum (including dependents) in 2005. Asylum seekers are a heterogeneous population. From 2003 to 2005, the UK received asylum applications from over 50 different countries, although the largest numbers came from Iraq, Iran, Eritrea and Somalia [2]. Many are from areas of political unrest and armed conflict and have experienced war, trauma and uncertainty about their family and friends [3,4]. Many will be experiencing physical and/or psychological health problems [5,6]. It is against this backdrop that health care professionals need to be able to provide appropriate and timely health care, meeting the needs of this culturally and spiritually diverse population.

In the UK, once an individual has made an application for asylum, they are eligible for support from the Government-run agency, the National Asylum Support Service (NASS) while their application is considered. As well as providing a small amount of money for subsistence, NASS also provides accommodation at various Local Authority council sites across the UK. At the end of 2005, there were an estimated 51,000 asylum seekers receiving NASS support, of which 35,140 were being supported in NASS dispersal accommodation in locations throughout the UK [2]. These sites are predominantly inner city, urban areas located within areas of socio-economic deprivation and include inner London, Newcastle, Birmingham, Liverpool and Manchester. While Glasgow is the only Scottish site receiving asylum seekers under this Government dispersal programme, it houses the largest number of asylum seekers of any Local Authority in the UK (5340 as of December 2005) [2]. Like other areas of the UK, asylum seekers have been housed in areas of socio-economic deprivation. Thus, findings from Glasgow are of immediate relevance to other areas of the UK. While the asylum seeking population in Glasgow is heterogenous, with over 40 countries of origin represented, 40% come from 4 countries: Pakistan; Turkey; Somalia; and Iran.

Asylum seekers and refugees are entitled to free health care under the National Health Service (NHS), although they must possess an HC2 exemption certificate, and to be registered with a general practitioner (GP) [6]. The HC2 exemption certificate is part of the NHS Low Income Scheme. It assists individuals on low incomes and entitles them to help with NHS charges such as prescriptions and dental treatment.

Previous studies have indicated that asylum seekers face a range of barriers when seeking health care, including access to interpreters [5-7], language barriers during the consultation [8,9], difficulty in accessing dental care [10], problems obtaining appointments [11-14] and different expectations of health care [13,15-17]. However, it is unclear if the NHS is becoming more responsive to the needs of asylum seekers or if it is becoming more adaptable to providing care for asylum seekers, particularly when the UK has such an explicit primary care gate-keeper model of health care organisation [18].

Interpreters also face problems relating to the process of interpreting consultations for asylum seekers. In particular, interpreters have reported tensions between acting as a dispassionate interpreter of the consultation and adopting the role of patient advocate and difficulties in translating medical terminology to patients [9,17,19,20]. However, good communication between asylum seeker, patient and health care professional is essential, with good language concordance found to lead to better outcomes from the consultation [8].

We report here on two related studies that sought to address some of these issues. Utilising focus groups and interviews with asylum seekers, the studies aimed to identify the barriers and facilitators to accessing health care, both medical and dental, and to explore the health care needs and beliefs of asylum seekers living in one part of the UK.

## Methods

### Setting

The studies were located in Glasgow, Scotland, which has received asylum seekers under the NASS dispersal programme since 2000. Two areas of Glasgow were selected, both with substantial numbers of asylum seekers living in the local community. In one area, where focus groups were conducted, the local primary care organisation had employed a dedicated asylum support nurse to facilitate GP registration, to conduct health checks and to act a conduit into primary care in general. This was not available in the area where the interviews were conducted.

### Recruitment and study participants

Recruitment was conducted through community-based groups, who facilitated access to members of the asylum seeking community either through direct introductions or by allowing the research team access to meetings attended by asylum seekers. In some cases, communication was facilitated through an interpreter. Individuals expressing an interest in the study received written materials, in their own language where possible, explaining the purpose of the study. These individuals were then followed-up and all individuals who agreed were recruited into the studies. Sampling was initially purposive in its approach, in that the research was located within two areas housing asylum seekers from a variety of countries of origin, including Iran, Sri Lanka, Somalia and other African countries and that it was hoped to recruit asylum seekers based on a range of characteristics, including gender, age and country of origin. However, given the vulnerability of the population of interest, it was not possible to directly recruit individuals by these specific characteristics. Thus, the sample was, in essence, of convenience but which did exhibit a range of characteristics, mainly due to the heterogeneous nature of the asylum seeking population itself (Tables 1, 2, 3).

**Table 1:** Countries of origin of the respondents.

	**Number of respondents**
**African Region**^a^	
Algeria	1
Democratic Republic of Congo (DRC)	7
Republic of Guinea	1
Zimbabwe	1
	
**Eastern Mediterranean Region**^a^	
Afghanistan	2
Iran	4
Lebanon	1
Morocco	1
Pakistan	1
Syrian Arab Republic	1
Somalia	8
	
**European Region**^a^	
Albania	1
Azerbaijan	1
Russian Federation	7
Turkey	7
	
**South-East Asia Region**^a^	
Sri Lanka	8

a. WHO regional classifications.

**Table 2:** Demographics of focus group participants.

	Focus Group	Gender	Country of Origin	Length of time in the UK
A	Farsi	Female	Iran	-
B	Farsi	Male	Afghanistan	1 yr 6 m
C	Farsi	Female	Afghanistan	2 yr
L	Farsi	Female	Iran	1 yr
A	Turkish	Male	Turkey	2 yr
N	Turkish	Male	Turkey	3 yr
J	Turkish	Male	Turkey	3 yr 6 m
M	Turkish	Male	Turkey	1 yr 9 m
H	Turkish	Male	Turkey	2 yr 6 m
F	Turkish	Female	Turkey	-
S	African	Female	DRC	3 yr 7 m
F	African	Female	DRC	4 m
FK	African	Female	DRC	-
T	African	Female	DRC	-
C	African	Male	DRC	2 yr
D	African	Female	Republic of Guinea	1 yr 7 m
G	Russian	Female	Russian Federation	-
V	Russian	Male	Russian Federation	-
Y	Russian	Female	Russian Federation	-
N	Russian	Male	Russian Federation	-
S	Russian	Female	Russian Federation	-
B	Russian	Female	Ukraine	-
H	Russian	Male	Ukraine	-
M	Women's	Female	DRC	2 yr
SM	Women's	Female	Morocco	2 yr
SD	Women's	Female	-	3 yr
E	Women's	Female	Zimbabwe	1 yr 6 m
N	Women's	Female	Sri Lanka	2 yr 6 m
F	Somali	Male	Somalia	-
SA	Somali	Female	Somalia	-
N	Somali	Female	Somalia	-
SU	Somali	Female	Somalia	-
Z	Somali	Female	Somalia	-
A	Somali	Female	Somalia	-
H	Somali	Male	Somalia	-
M	Somali	Female	Somalia	-

**Table 3:** Demographics of those interviewed.

	Gender	Age	Country of Origin and Religion	Length of time in the UK
R1	Male	51	Sri Lanka (Tamil Hindu)	4 yrs 6 mths
R2	Male	55	Sri Lanka (Tamil Hindu)	4 yrs 2 mths
R3	Male	40	Iran (Zoarastrian)	2 yrs 6 mths
R4	Male	35	Iran (Zoarastrian)	5 yrs
R5	Male	39	Sri Lanka (Tamil Christian)	5 yrs
R6	Female	22	Syria (Muslim)	4 yrs
R7	Male	47	Azerbaijan (Jehovah's Witness)	3 yrs
R8	Female	21	Turkey (Muslim)	4 yrs 6 mths
R9	Female	42	Albania (Muslim)	5 yrs
R10	Male	50	Algeria (Muslim)	4 yrs
R11	Male	45	Pakistan (Muslim)	4 yrs
R12	Female	30	Lebanon (Muslim)	2 yrs 3 mths
R13	Female	20	Sri Lanka (Tamil Hindu)	5 yrs
R14	Female	21	Sri Lanka (Tamil Hindu)	5 yrs
R15	Female	57	Sri Lanka (Tamil Hindu)	5 yrs
R16	Female	46	Sri Lanka (Tamil Hindu)	4 yrs 7 mths

R9 to R12 and R13 to 15: Conducted as a group interviews.

Participants were at varying stages of the asylum seeking process with a few having reached full refugee status. However, we refer to the population throughout as asylum seekers.

### Data collection

Two methods of data collection were employed: focus groups facilitated by members of the asylum seeking community and one-to-one or group interviews, conducted through an interpreter.

Focus group topic guides and interview schedules were developed following a review of the literature and other work in this area, for example work located in Sunderland [14]. The guides covered a range of topics including their use of health services; barriers and facilitators to accessing care; use of secondary care services; use of dental services; experience of translators; and previous experience of health care in their own country.

#### (i) Focus groups

Focus groups involved the participation of a group of 5 asylum seekers (4 males and 1 female), who volunteered to be trained as focus group facilitators. This training was led by MH and covered issues such as: managing discussion with a topic guide; awareness of group dynamics, handling sensitive or difficult questions; and maintaining confidentiality.

Six focus groups were conducted between winter 2003 and summer 2004, each with 5 to 8 participants and lasting 1.5 to 2 hours. These were constructed according to ethnic (e.g. Turkish group) or language (e.g. Farsi group) similarities and were conducted in the language appropriate to that focus group: Farsi; Turkish; French and Lingala (an African language spoken in the Democratic Republic of Congo); Swahili (for the Somali group); and Russian. One all woman group was conducted in English by MH, assisted by the female facilitator. As well as facilitating the focus groups, these volunteers also assisted in the process of translating the focus group transcripts back into English, for transcribing.

#### (ii) Interviews

Sixteen individuals were interviewed in the summer of 2005 by RC: 9 one-to-one interviews and 2 as joint narratives, the first with 4 participants and the second with 3 members of the same family. All but one were conducted through a professional interpreter: thus, the researcher asked a question which was simultaneously translated into the appropriate language. The interviewee's response was then translated back into English and it was this that was transcribed, as described below. Interviews lasted about 1 hour and were conducted at a venue chosen by the interviewee.

The concept of written consent was explained to all participants before the focus group or interview commenced and written consent obtained.

### Analyses

Focus groups and interviews were taped and transcribed verbatim. In the case of the focus groups, group facilitators assisted MH to translate the tapes back into English, before being transcribed. For interviews, the interviewer's questions and translators English responses were translated.

Analysis was facilitated using the Framework method, which involved the systematic process of identifying, charting and sorting the data according to key issues and themes [21]. The research team read the first five transcripts to identify broad themes, guided by the topic guide and interview schedule. These were discussed and refined, leading to a coding schedule applied to all transcripts. Analysis was iterative, with broad themes identified first (e.g. access; interpreters), then broken down into sub-themes (e.g. making appointments; confidence in interpreter) [22]. A constant comparative approach was used throughout, whereby the codes and transcripts were continually re-assessed and re-interpreted [23]. Identified themes were compared across the data and interpretations discussed within the team. In the case of focus group transcripts, analyses sessions were also held with the facilitators in order to enhance the research team's understanding and interpretation of the data. Identified themes in the focus groups and interviews were compared, again to identify common and discrepant themes. Quotations were chosen to illustrate particular points and are identified by an anonymised code, as well the gender and country of origin of the respondent and whether a focus group or interview.

### Ethical Approval

Both studies received ethical approval from the NHS Greater Glasgow Primary Care Research Ethics Committee.

## Results

A total of 52 individuals participated in the research: 16 were one-to-one or group interviews; the remaining 36 participated in one of six focus groups. Respondents came from a wide range of countries (Table 1). Their ages ranged from 20 – 57; 31 were female, 21 male and most had been in the UK for at least 3 years (Tables 2 &3).

We have organised the findings by the 6 major themes that emerged from the analysis of both the focus groups and the interviews. These are discussed in turn.

### Access to health care

Most asylum seekers arriving in Glasgow received written information from the health board telling them how and where to register with a general practice. In one site, an Asylum Support Nurse had been employed to contact asylum seekers, go over the registration process with them, conduct a health check then inform them which surgery to register with. As a result, most of these respondents (who were the 36 focus group participants) were registered with a GP and the prevailing theme that emerged here was one of feeling welcomed and cared for as a result of this process.

This positive experience was not shared by all asylum seekers. A few were concerned that they had been told to register with a GP who was not the nearest one to where they were staying. Others, particularly those who had arrived at the beginning of the dispersal process, had either received postal information or no information at all. Some thought that the information they had received had come directly from NASS rather than from the local NHS Health Board.

Many experienced difficulties getting timely appointments with their GP. Respondents often considered their symptoms as an emergency, e.g. flu symptoms, stomach pain, and wanted to be seen quickly. There was frustration when they had to wait several days for an appointment, especially when symptoms improved before the consultation. This was a particular issue when children were involved:

*"... when you take the kid there she is crying but they would tell you to take her back home 'til tomorrow at ten o'clock. But when you take her tomorrow at ten o'clock the stomach *[pain] *has stopped." (F, Male, Somali Focus Group)*.

Some circumvented this problem by calling an ambulance or going directly to the hospital. One explanation for their response to such symptoms was their perception of the potential severity of outcome. In describing a situation where his child had flu-like symptoms and a blocked nose, one man said:

*...at the hospital they will tell you it's not the right time to give him medication its only flu ... On our side flu is a big issue because the kid may die. If the kid cannot breathe normally then the kid will die." (H, Male, Somali Focus Group)*.

Most reported positive experiences regarding the care provided by their GPs, even when they re-told incidents where they felt care had not met their expectations, e.g. hoping for a referral to secondary care but instead receiving a prescription. However, GPs were often perceived as not being specialised, with this impacting on respondents' behaviour. For example if the problem was deemed to be an emergency or requiring a specialist, some would go directly to hospital. Others commented on the lack of continuity, seeing a different doctor each time they attended the surgery. This was a particular problem at a branch surgery where locums were often used:

*"They *[locums] *are finding it difficult to understand. We have to explain everything and then they have to go deep into the file to give us medicine whereas Dr X will know automatically." (R16, Female, Sri Lanka, Interview)*.

However a few respondents expressed surprise when they discovered they could ask for a particular doctor if they wished and that they had a specific GP whose care they were under.

Many had experience of secondary care, as a hospital in-patient, outpatient or through accident and emergency departments. People again reported largely positive experiences, although there were problems accessing interpreters (see below). There was also surprise and disappointment at the length of waiting times both for hospital appointments and at accident and emergency.

*"I had to wait six months for a hospital appointment. I asked about this because I was worried but the doctor said oh it's not a serious problem and that we should wait." (C, Afghani Female, Farsi Focus Group)*.

Most respondents had attended a dentist. However, the experience of finding and registering with a dentist was different to that with GPs. Several couldn't remember if the initial information packs had contained any information on dental services. Some got their information from their GP; some had received a letter telling them to register with a dentist, but others didn't know where it had come from. Some reported difficulty finding a dentist who would treat asylum seekers. There was little to suggest that respondents attended dentists for regular check-ups, instead appearing to use them when there was a clinical need.

### Experience with interpreters

#### (i) Provision

Interpreting was a major issue. In general, the provision of interpreters in primary care appeared to be well organised and fairly reliable. Respondents spoke of surgeries organising interpreters both for GP consultations and for appointments in secondary care. However, the provision of interpreters during in-patient stays was less reliable, with patients lacking interpreters at key points in their hospital stay, e.g. when waking up after an operation or during in-patient stays.

*The first day in hospital was fine because there was an interpreter there for me. But I was in for four days and the next four days there were no interpreters. Doctors were asking me things but I couldn't understand and I wanted to ask them things but they didn't understand. (A, Iranian Female, Farsi Focus Group)*.

Data from one focus group discussion suggested that interpreting services are also required in pharmacies, to facilitate questions about medication names and regimens.

The use of interpreters was less common at the dentist compared with the GP. This appeared to be due less to a lack of provision and more to a perceived lack of need:

*We don't need to talk to him, he checks our teeth. (R15, Female, Sri Lanka, Interview)*.

#### (ii) Quality

A number of respondents, particularly those from Sri Lanka, reported difficulties obtaining appropriate interpreters. Often, they were sent an Indian Tamil, which led to difficulties in communication due to the differences between Sri Lankan and Indian Tamil. Difficulties speaking English meant that this problem was not properly addressed.

There were times when respondents felt that interpreters were not fulfilling their duties appropriately, for example by not re-telling their story to the doctor correctly. Another respondent felt uncomfortable with interpreters within the consultation, arguing that you couldn't be sure that it was confidential. In one focus group, there was the view that interpreters were not trained to deal with health issues or medical terminology. Yet there was a demand for this service as evidenced by one respondent who explained that although his family could interpret for him, he preferred a professional who could help with medical terms.

An alternative view came from a respondent who was also an interpreter. He suggested that people expect their interpreter to act as an advocate. However, interpreters are trained to interpret literally. He suggested that clearer instructions were required, to reassure patients that interpreters are bound by a code of confidentiality but also that are unable to play an advocacy role.

### Experiences of other health care staff

Respondents had experienced a range of other health care staff including health visitors, other nursing staff, receptionists and opticians. Most experiences were reported as positive:

*They are all very very good. They are very patient and so far they are tolerant (R9, Female, Albania, Interview)*.

However, several respondents spoke of times when they felt discriminated against or treated badly because they were asylum seekers. One spoke of an incident he witnessed in secondary care when a consultant was derisory towards a patient for being an asylum seeker; another felt that a receptionist used to discriminate against asylum seekers when they were trying to make appointments. One respondent felt continually discriminated against, citing that health care professionals had stopped coming to his house when they found out they were asylum seekers. Others echoed this feeling of discrimination and isolation:

*Sometimes you feel left out and think being an asylum seeker, you are different (E, Zimbabwean Female, Woman's Focus Group)*.

Asylum seekers who had been in contact with health visitors were particularly positive about the help and support they had received over a range of issues, both health and social. Health visitors instilled a sense of confidence and confidentiality, perhaps because they came to their homes. One respondent offered a note of caution over how much this professional group can be expected to achieve:

*I've noticed people being seen by health visitors and are quite happy about them, about health visitors to go to them and see them because they think somebody is paying attention to me so they really appreciate that but then the problem with that is that they expect too much from the health visitor. ...... They don't understand that the health visitor's hands are often tied, they feel that is a disappointment. (R4, Male, Iran, Interview)*.

### Barriers to care

Respondents rarely spoke directly about barriers to care, however several became apparent during analysis. The first was language, particularly in situations where there was no interpreter, e.g. when phoning the surgery to negotiate for an appointment. Access to medication was another barrier, with a number of respondents citing times when they were expected to pay for painkillers, such as paracetamol, over the counter rather then receiving a prescription. This was a significant cost for families where young children often needed childhood cold and flu remedies, particularly when the family had received an HC2 certificate and expected to be exempt from paying.

E:*Then after she *[GP] *gave a prescription she said "You should go and buy this for yourself, because if I give you a prescription it is expensive. It is cheaper if you buy it yourself...." Some things you have to buy, what's the use of the medical certificate *[HC2] *then?*

S:*Mm yes, I experienced this ....*

E (to S):*You understand? (Zimbabwean and Moroccan Females, Woman's Focus Group)*.

Another barrier was access to specialists in secondary care. There were two issues: perceptions that the GP was not referring them to see a specialist and waiting times when they were. Several felt that there were situations where they would prefer to be referred to a specialist but where the GP was either reluctant or took other action e.g. prescribing medication. One respondent commented that he felt the GP actively avoided further referral. Many of the respondents also commented on the long waiting times to see a specialist, a view articulated most bleakly here:

*I am very sorry, in this country one maybe die before he has received his appointment. (R10, Male, Algeria, Interview)*.

There was a view from some that this could be attributed to the fact that they were asylum seekers. However others, particularly those with a better command of English, took the view that waiting lists affected everyone in the UK.

*I think it is a common problem for everybody. It's not that easy to get a specialist in this country, ... (R3, Male, Iran, Interview)*.

*The waiting list was a bit unbelievable for me because the image we had under medicine in the UK was sort of different. Now I know why people refer to the private sector.......... So people on the NHS have just got long waiting lists, not just for me, for everybody. (R4, Male, Iran, Interview)*.

### Needs/gaps in services

Data highlighted the need for better access to mental health/psychology services. Many respondents talked of extreme anxiety to the point of frequent panic attacks and feelings of sadness and loss. However many seemed unaware that any help could be offered for this and felt it was somehow inappropriate or hopeless to discuss such matters with the GP:

Sensitive health issues, such as rape and HIV, were unsurprisingly not discussed in this context but data from a related study (to be published separately) which interviewed health care professionals caring for asylum seekers suggests there are gaps in services here linked to both perceived low disclosure due to lack of trust and lack of specialist referral services.

Provision of appropriate health information was also an issue, with a lack of appropriately translated health information leaflets. There was also a lack of knowledge about health promotion and health screening programmes. Cervical screening was discussed in the woman's group, where there was confusion over the frequency over smears, fear and embarrassment over the procedure and a view that it was unnecessary when one felt well.

Few respondents knew about the provision of out-of-hours primary medical care, with only one respondent indicating that they had used the primary care emergency service out with routine surgery hours. Where medical care was required at night, respondents either attended A&E or called an ambulance. While we had no information on the clinical condition that required these visits, other than the patients' recounting of the event, some patients did talk about being admitted to hospital suggesting that their condition was of a more serious nature. In other situations, the patient was sent home again and told to attend their GP in the morning. This led to the following comment:

*Sometimes at night the GP is not open so that is why we have to go to hospitals and they say you should wait until morning and be sent back, it's not good. I don't know, we could die if we wait until morning and something opens. (R8, Female, Turkey, Interview)*.

### Comparison with health services in their country of origin

Respondents came from countries with health services ranging from the well developed and modern (e.g. Syria, Iran) to war areas where health care had completely broken down (e.g. Tamil-held Sri Lanka). A common feature to all these settings, however, was the absence of a primary care system impacting on their views of health care here, in particular expectations regarding access to specialists and a view that GPs were not specialised enough to deal with their problems.

*Sometimes when the doctors or GPs aren't specializing in the thing, they just say you are OK. ....... They think that you are OK and you think that you are not OK. (R6, Female, Syria, Interview)*.

In general, all respondents compared the UK NHS favourably with health care systems in their own country, particularly where internal conflict had led to destruction of existing health care systems. Those from countries with more developed health systems were more cautious. While positive about health care in the UK, many were used to systems where one could access a hospital-based specialist immediately, albeit having to pay for that care.

Access to antibiotics was also raised as, in some countries, antibiotics were readily available on payment from a pharmacy. This led to expectations that antibiotics would also be readily available in the UK and disappointment when they were not prescribed.

*...some people are not happy with the doctor because oh they didn't give us antibiotics... (R3, Male, Iran, Interview)*.

Thus, the type of health care system that they were used to clearly impacted on their expectations of health care in the UK.

## Discussion

This paper describes the views of asylum seekers regarding access to and knowledge of health care in the UK. The findings come from two studies, both employing qualitative methods and separated in time by approximately one year. The first study utilised focus groups with members of the asylum seeking community trained to act as facilitators. This study was located in one area of Glasgow, which employed a dedicated asylum support nurse; thus, we felt it important to determine if the issues raised were pertinent to asylum seekers living in areas without such a support worker. However, the training and support required by the facilitators made it difficult to reproduce elsewhere. In addition, it was felt that the use of another methods of data collection, namely interviews, would act as a form of triangulation and strengthen the overall findings, as utilised in a previous study on young people's experiences of their social world [24].

The study confirmed several findings from other studies, for example access to GPs for routine appointments was often problematic, especially for conditions perceived to be emergencies, as previously reported ([13,14,25]. In some cases, respondents said they would attend accident and emergency departments or call an ambulance instead. We do not have data on accident and emergency visits or ambulance calls, so cannot corroborate this view. However, our work also highlighted a lack of awareness of primary medical care out with routine surgery hours, which suggests that such approaches are used by asylum seekers when they are unsure where else to seek help or cannot access help when required. Another explanation, which emerged during this study, was the perception that such symptoms may deteriorate into more serious conditions if not treated immediately. This may be due, in part, to the illness profiles in their countries of origin, where symptoms regarded as self-limiting the West may indeed develop into more serious illness.

Once asylum seekers saw a GP, the experience was generally positive as was encounters with other health care professionals, especially health visitors. The role of the Asylum Support nurse, which was unique to one of the study areas, generated good outcomes in terms of registration levels, general health checks and importantly fostered trusting relations between patients and services. However, there was a broad perception that GPs could not be knowledgeable enough to deal with all the conditions that they see in practice and that health care ought to be provided by specialists. This may reflect the countries of origin of the participants, which do not have systems of generalists providing health care [26]. Other areas of difficulty included access to interpreters in secondary care, lack of knowledge of health promotion and screening programmes, access to primary care services out with surgery hours and to specialists in secondary care. Again, some of views are likely to be coloured by the health care system that they were used to in their own country. While these findings mirrored those from other studies [14,16,17], they also reflect barriers experienced more generally by other minority ethnic populations [27]. Thus, while some issues are specific to individual populations of asylum seekers, addressing the more generic issues are likely to improve the quality of care for a wider range of patients.

The finding that many thought that their letter informing them about GP registration came from NASS may have implications about how they then view health care in the UK. It is possible that they associate the initial registration visit to the GP with their asylum application and this may also explain why they express worries about the confidentiality of encounters involving interpreters. Written materials, given to them on arrival, were not used as a source of information when required later. This suggests the need to develop different approaches to giving people information about health, health care systems and how to use them.

Interpreting services were generally well provided in primary care, suggesting that some of the barriers previously highlighted have been resolved [5,6]. However, language barriers within the consultation remain [8,9,19]. In Glasgow, a particular problem was that of interpreters speaking a different dialect from the asylum seeker e.g. Sri Lankan versus Indian Tamil. More generally, the use of interpreters needs to be improved within the secondary care setting and the role of interpreters within the consultation also needs to be clarified and developed.

Our work confirmed previous work that specialist help was regarded as important in medical settings and that people want a proactive, empathetic interpreter rather than someone who just translates verbatim [28]. However, one interviewee, who had acted as an interpreter for others, offered a valuable insight into the dilemma that interpreters, particularly professional interpreters, face in that they are trained to act as literal conduits for information rather than acting as a patient advocate. This is in agreement with recent findings from Greenhalgh et al, who reported that interpreters saw their professionalism as being closely tied to the accuracy of their translation and their avoidance of involvement in the consultation [19]. In contrast, interpreters employed as link workers or as part of community organisations are more likely to see their role as that of patient advocates [9]. Thus, a review of the role of interpreters in the consultation could see them take on a more patient-centred advocate role than may currently be the case, ensuring that patient-doctor communication within the consultation is optimised [17]. While this has implications for the future training of interpreters, there was no evidence that such an approach was being taken.

While the use of focus groups and interviews led to the identification of generic issues, differences were apparent in the extent to which participants were prepared to be critical of the health care system. Interviewees were generally positive about their own personal experiences. Negative experiences were usually about "someone else". This public versus private face is understandable, given their circumstances. It was, however, less apparent in the focus group setting, where participants were more confident and willing to discuss their own negative experiences. This mirrors the experience of Michell [24], although in that research it was the interview setting which allowed those excluded from focus group discussions to articulate their views and feelings more strongly. These findings highlight the care that needs to be taken when interpreting findings from only one methodological approach and when considering whether the health service is currently meeting all the needs of this population.

These studies did have limitations. The trained focus group facilitators were known to some of the participants and all but one interview was conducted through an interpreter. In some cases, the interpreter was well known to the interviewee and was clearly knowledgeable about their case. Both of these factors could have had a positive effect in terms of trust and rapport but, in the case of the interpreted interviews, it was at times difficult to know if we were obtaining the interviewee's views or a distilled view from the interpreter.

There was no opportunity to purposively sample at an individual level and the countries of origin of respondents was partly dictated by the settings in which the research was conducted, for example one area had a large population of Sri Lankan Tamils. However, similar issues arose in all the focus groups and interviews, suggesting that these are generic issues affecting all asylum seekers, regardless of their country of origin. Fear and anxiety about their asylum application and the socio-economic situation in which they find themselves have as great an impact on their experience as their cultural background [29]. This must be borne in mind when trying to develop ways of working with asylum seekers and prioritising needs.

## Conclusion

The studies reported here add to our understanding of the issues facing asylum seekers when they access NHS care. Our research confirms previous important findings regarding issues of access to timely health care and the role of interpreters within the consultation. Interpreting services should continue to be strengthened and developed, with particular attention paid to their role in secondary care and developing the role of the interpreter as a patient advocate. In addition, we suggest that attention also needs to be paid to asylum seekers' understanding of how the NHS works, in particular the role of GPs and referral to specialists. These areas could the target of initiatives to increase their awareness and understanding, for example the use of patient advocates to explain how the NHS works or to help asylum seekers access care out with normal surgery hours. In this way, care for an important minority ethnic population can continue to be developed and improved.

## Competing interests

The author(s) declare that they have no competing interests.

## Authors' contributions

COD led the design of the focus group study; COD, MH and KM led the design of the interview study. MH conducted the focus group training, organised the focus groups and worked with the facilitators; RC conducted the interviews. MH and COD analysed the focus group data and all the authors were involved in the analysis of the interview data. COD drafted the manuscript, with comments from the other authors. All authors saw and approved the final version.

## Pre-publication history

The pre-publication history for this paper can be accessed here:


